# Chronisch nicht-bakterielle CNO in der Adoleszenz: Eine therapeutische Herausforderung der Rheumatologie

**DOI:** 10.1007/s00393-026-01778-5

**Published:** 2026-02-02

**Authors:** Michael Schmidt, Claus Schneider, Martin Scheer

**Affiliations:** 1 Klinik für Rheumatologie und klinische Immunologie, Johannes Wesling Klinikum Minden/Universitätsklinik der Ruhr-Universität Bochum, Hans-Nolte-Straße 1, 32429 Minden, Deutschland; 2 Universitätsinstitut für Radiologie, Neuroradiologie und Nuklearmedizin, Johannes Wesling Klinikum Minden/Universitätsklinik der Ruhr-Universität Bochum, Minden, Deutschland; 3 Klinik für Mund‑, Kiefer- und Plastische Gesichtschirurgie, Johannes Wesling Klinikum Minden/Universitätsklinik der Ruhr-Universität Bochum, Minden, Deutschland

**Keywords:** Kiefer, Adoleszenz, MRT, Pamidronat, Adalimumab, Jaw, Adolescence, MRI, Pamidronate, Adalimumab

## Abstract

Unsere Kasuistik zeigt die Herausforderungen in der Therapie einer chronisch nicht-bakteriellen Osteitis/Osteomyelitis (CNO) des Kiefers auf und unterstützt die aktuellen Empfehlungen zur Diagnose und Therapie, welche neu definierte medikamentöse Behandlungsstrategien priorisieren. Dabei ist die Implementierung regelmäßiger Reevaluationen –  sofern erforderlich auch mittels Magnetresonanztomographie – für die gewünschte Treat-to-Target-Strategie essenziell. Die Patientenbetreuung sollte in Zentren mit interdisziplinären Ausrichtungen und Erfahrungen in der Behandlung der CNO erfolgen.

## Abstract (en)

Our case study highlights the challenges in treating chronic non-bacterial osteitis/osteomyelitis (CNO) of the jaw and supports the current recommendations for diagnosis and treatment, which prioritize newly defined drug treatment strategies. The implementation of regular reevaluations—including magnetic resonance imaging (MRI) if necessary—is essential for the desired treat-to-target strategy. The patient care should be carried out in centers with an interdisciplinary approach and experience in the treatment of CNO.

## Einleitung

Die chronisch nicht-bakterielle Osteitis/Osteomyelitis (CNO) des Kiefers mit typischer Manifestation in der 2. Dekade tritt sowohl solitär, aber auch als Teil einer multifokalen CNO, oft ohne, seltener als Variante der SAPHO-Erkrankungsgruppe (*Skin-Bone-Disease*) mit Hautbeteiligung in Erscheinung.

Initial von der Spongiosa ausgehend, können im Verlauf sowohl die Kortikalis, das Periost und das umgebende Weichteilgewebe in den entzündlichen Prozess involviert werden, was mittels Magnetresonanztomographie (MRT) dann als Knochen- bzw. Knochenmarködem und Weichteilödem mit positivem Kontrastmittel-Enhancement dargestellt wird. In der Computertomographie (CT) kommen intraossär Osteolysen neben Sklerosierungen mit unscharfer Kortikalisbegrenzung sowie periostal lamelläre oder auch solide reaktive Knochenneubildungen zur Darstellung. Im Gegenzug können diesbezüglich rückläufige und letztlich normalisierte Befunde als Kriterien der Remission bewertet werden.

Nach Ausschluss tumoröser, traumatischer oder infektiöser Differenzialdiagnosen zielt die Therapie auf eine Beendigung des autoinflammatorisch generierten proliferativen Prozesses ab.

Eine Triggerung durch Veränderungen der Mundflora ist Gegenstand der Diskussion. Antibiotika sind, wie auch bei unserer Patientin, nicht selten initialer Bestandteil des Behandlungskonzepts [1].

## Vorgeschichte

**Abb. 1:**
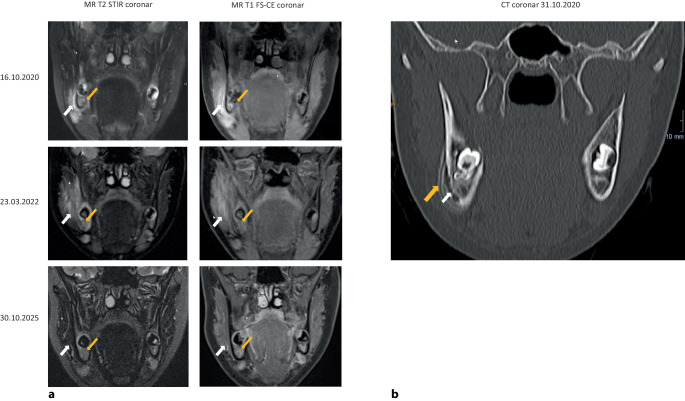
**a***Obere Zeile* Die MR-T2-Sequenz koronar mit STIR-Fettunterdrückung (MR T2 STIR cor; *linke Spalte*) vom 16.10.2020 zeigt ein Knochenödem (*Pfeil*, *gelb*) und ein unmittelbar angrenzendes Weichteilödem (*Pfeil*, *weiß*). In der MR-T1-Sequenz mit Fettsättigung koronar nach Kontrastmittel (MR T1 FS-CE cor; *rechte Spalte*) kommen das Knochenödem und das Weichteilödem kontrastreicher und ausgedehnter zur Darstellung (entsprechende *Pfeile*, *gelb* bzw. *weiß*). *Mittlere Zeile* Die MRT vom 23.03.2022: Die MR-T2-STIR cor zeigt im Vergleich zur Voruntersuchung weiterhin ein deutliches Knochenödem sowie eine flächige Zunahme der Weichteilödemausdehnung. Die MR-T1 FS-CE cor zeigt hier zusätzlich ein stärkeres KM-Enhancement der knöchernen Strukturen und der umgebenden Weichteile. (*Pfeilmarkierungen* analog obere Zeile). *Untere Zeile* MRT vom 30.10.2025: MR T2 STIR cor (*linke Spalte*) und MR T1 FS-CE cor (*rechte Spalte*): Die knöchernen Strukturen, das Periost und umgebende Weichgewebe zeigen deutlich rückläufige Befunde. Hier finden sich weder in der STIR-Sequenz noch in der KM-Serie signifikant pathologische Signale. (*Pfeilmarkierungen*, analog obere Zeile). **b** CT vom 30.10.2020: Die CT zeigt eine Arrosion der Kompakta (*weißer Pfeil*) sowie eine lamelläre Periostreaktion dort angrenzend (*gelber Pfeil*)

Im *Oktober 2020* erfolgte die Vorstellung der damals 13-jährigen Patientin beim Hauszahnarzt zur Abklärung einer Schwellung des rechten Unterkiefers. Es folgten eine 20-tägige Antibiotikatherapie mit Clindamycin sowie eine erste bildgebende Diagnostik mittels MRT (Abb. 1a) und CT (Abb. 1b) sowie eine histologische Untersuchung (externe Mitteilung, s. unten).

Im *Dezember 2020 *stellte sich die Patientin erstmalig in der Klinik für Mund-Kiefer-Gesichts-Chirurgie (MKG) des Johannes Wesling Klinikums (JWK)/Universitätsklinik der Ruhr-Universität Bochum (RUB) mit dem Leitsymptom *mehrmonatiges Druckgefühl und Schwellung* im Bereich des *rechten Unterkiefers *vor. Die Entfernung der letzten Milchzähne bewirkte nur vorübergehende Linderung. Im Rahmen der klinischen Untersuchung fanden sich ein aufgetriebener Unterkieferknochen rechts, eine Schwellung der rechten Wange, jedoch keine Rötung, keine Abszedierung und keine Einschränkung der Mundöffnung. Die weitere Untersuchung erbrachte keine Sensibilitätsstörungen, es fand sich eine regelrechte Spitz-Stumpf-Unterscheidung im Innervationsgebiet des N. alveolaris inferior rechts.

Die *Bildgebung mittels digitaler Volumentomographie (DVT)* zeigte eine deutliche Auftreibung der Pars horizontalis des rechten Unterkiefers nach lateral sowie knöcherne Defektzonen im mittleren Anteil mit einer Ausdehnung von ca. 8 mm nach alio loco durchgeführter Biopsie im Oktober 2020 (s. oben). Die Knochenbiospie ergab histologisch eine fibrosierende Inflammation ohne Nachweis einer bakteriellen Osteomyelitis nach Konsultation des osteopathologischen Referenz-Zentrums, Vivantes Klinikum Berlin-Friedrichshain.

Therapeutisch wurde eine Dekortikation mit modellierender Osteotomie des rechten Unterkiefers über einen intraoralen Zugang diskutiert, patientenseitig wurde Bedenkzeit erwünscht, der Eingriff aber letztlich nicht durchgeführt.

*Von Januar 2021 bis Februar 2022 *erfolgte – bei insgesamt undulierendem Verlauf – eine symptomatische Behandlung mit zum Teil längerfristiger Einnahme von NSAR (nichtsteroidale Antirheumatika) und bedarfsorientierter Anwendung von Glukokortikoiden (GC) im Dosisbereich zwischen 5 und 25 mg. Neben nahezu asymptomatischen Phasen berichtete die Patientin immer wieder auch von akuten Schmerzattacken, so dass sie mehrfach elektiv, aber auch notfallmäßig in der Klinik für MKG des JWK Minden gesehen wurde.

## Rheumatologische Abklärung

Im *Februar 2022* erfolgte dann die notfallmäßige Wiedervorstellung der Patientin in der Klinik für MKG des JWK Minden wegen einer *deutlich zunehmenden *aber relativ *schmerzarmen Schwellung *des *Unterkiefers *und des *Kieferwinkels rechts* sowie der rechten Wange, wobei zeitgleich eine *erheblich eingeschränkte Mundöffnung *auf nur noch ca. 10 mm auffiel.

Zudem lag eine milde Akne vor. Es zeigten sich aber weder Arthritis noch weitere Hautveränderungen, z. B. im Sinne einer palmoplantaren Pustulosis. Nach befristeter Antibiotikatherapie mit Ampicillin/Sulbactam schloss sich dann die Übernahme in die Klinik für Rheumatologie und klinische Immunologie (für Erwachsene) des JWK Minden an.

Im Rahmen der Laboruntersuchungen fanden sich normwertige Entzündungsparameter und ein ANA-Titer von 1:320. Die weitere Autoimmunserologie blieb unauffällig, HLA B27 war nicht nachweisbar.

Die *MRT *des *Gesichtsschädels* (Abb. 1a) zeigte ein Ödem der Spongiosa und der benachbarten Weichteile sowie eine ausgedehnte Kontrastmittelreaktion in diesem Areal als Korrelat der CNO im Bereich der Mandibula rechts. Eine *Ganzkörper-MRT* blieb ohne Nachweis weiterer Knochenödeme oder Gelenkergüsse.

## Diagnose

- *juvenile idiopathische Unterkieferosteomyelitis rechts/CNO der Mandibula rechts mit Akne als Teilaspekt eines SAPHO-Syndroms*

## Rheumatologische Therapie und Reevalutionen im Verlauf (Abb. 2)

Im *März 2022 *begannen wir daraufhin eine Therapie mit *Azithromycin* per os (p.o.) 500 mg täglich (tgl.), initial an 5 aufeinanderfolgenden Tagen, anschließend fortgesetzt niedrig dosiert 2‑mal/Woche über insgesamt 12 Wochen. Zeitgleich starteten wir eine orale *Sulfasalazin-*Aufdosierung über 3 Wochen auf 3‑mal 500 mg täglich sowie eine *Ibuprofen-Medikation *mit 600 mg 1‑0‑1 p.o., parallel hierzu medikamentöse Ulkusprophylaxe und Kontrolle der Folatspiegel/Folatsubstitution.

**Abb. 2:**
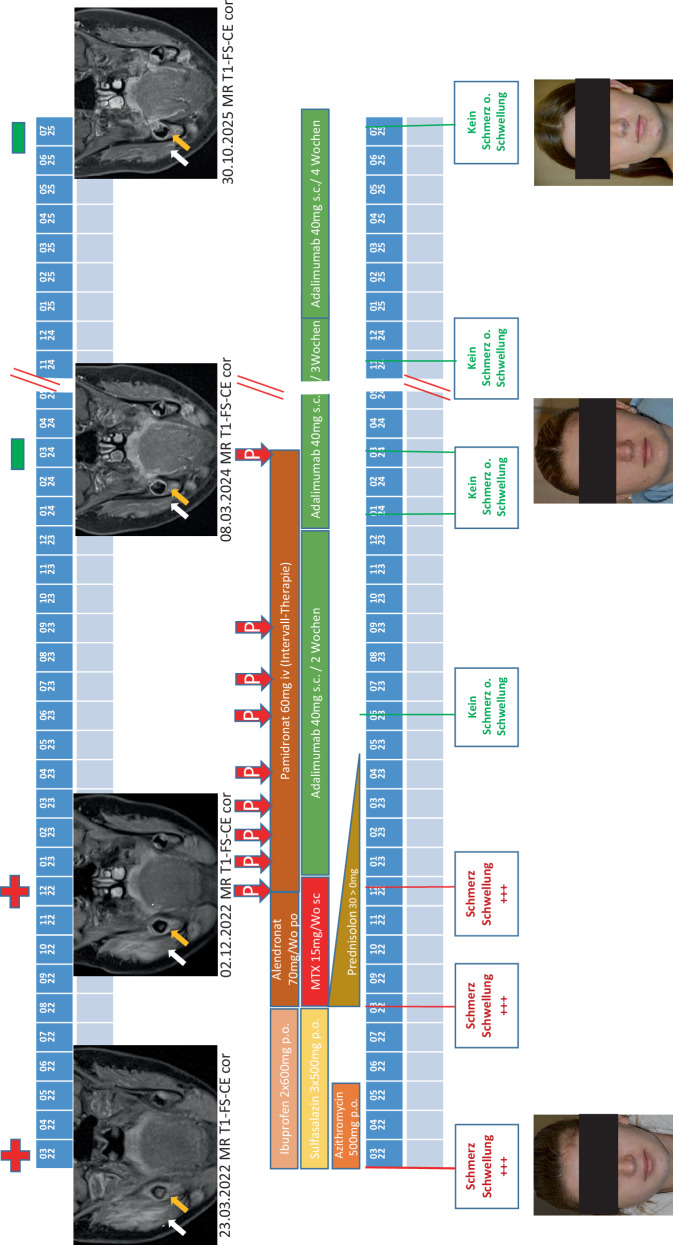
Im zeitlichen Ablauf schematisch parallel in 3 horizontalen Blöcken wie folgt dargestellt (*von oben nach unten*). *Kopfzeile* MRT-Untersuchungen mit graphischer Markierung + (pathologisch)/− (regredient) der Befundergebnisse (orientierend). Darstellung MRT-Verlauf (T1-FS-CE cor). *Gelbe Pfeile *Knochenauftreibung bzw. Knochenödem, *weiße Pfeile *Weichteilauftreibung/Ödem. Befunde im Verlauf deutlich rückläufig. *Mittelblock* Darstellung der relevanten Medikamente mit Dosierungen, Applikationen in zeitlicher Abfolge (*von links nach rechts*): zunächst Ibuprofen + Sulfasalazin + Azithromycin, dann Alendronat + Methotrexat (MTX) + Prednisolon, dann Pamidronat-Intervall-Therapie (*rote Pfeile mit KennbuchstabenP*) + Adalimumab. *Sockel* Die parallel erfassten Symptome und Fotografien der Patientin

Im *Juli 2022 *berichtete die Patientin über lokal *zunehmende Schmerzen* bei klinisch weiterhin deutlich sichtbarer Schwellung im Bereich der rechten Mandibula, woraufhin eine *Therapie-Umstellung *auf *Methotrexat (MTX) 15mg/Woche s.c.* (plus 5 mg Folat am Folgetag) initiiert wurde, in Kombination mit Prednisolon, initial 30 mg tgl. mit anschließend wöchentlicher Dosisreduktion in 5‑mg-Schritten bis zur Erhaltungsdosis von 5 mg tgl. Parallel begannen wir eine orale Bisphosphonat-Behandlung mit *Alendronat 70mg/Woche p.o*. in Kombination mit 20.000 IE Vitamin D3 einmal wöchentlich.

Im *Dezember 2022 *erfolgte dann die *Reevaluation *inklusive *Verlaufs-MRT. *Dort zeigten sich morphologisch *zunehmende* entzündliche Veränderungen in der Mandibula rechts, die nun bis an das rechte Kiefergelenk heranreichten und auch die rechtsseitige Kaumuskulatur involvierten. Ein Gelenkerguss war nicht erkennbar, aber eine leichte Größenzunahme submandibulärer Lymphknoten rechts.

Im *Dezember 2022 *nahmen wir daraufhin eine *Umstellung *der p.o.-Bisphosphonat-Therapie auf eine intravenöse (i.v.) Therapie mit *Pamidronat 60mg *vor – zunächst 6 Applikationen in 4‑wöchentlichem Abstand. MTX (15 mg/Woche s.c.) wurde bis zum Jahreswechsel 2022/2023 weitergeführt, dann beendet. Hierunter trat eine moderate Besserung der Beschwerden ein, ohne dass jedoch auf NSAR oder GC verzichtet werden konnte.

Im *Januar 2023*, unter Fortsetzung der 4‑wöchentlichen Pamidronat-Intervall-Gaben, erfolgte eine Erweiterung der Therapie mit *Adalimumab 40mg *alle 2 Wochen subcutan (s.c.).

Im *Juni 2023* fand sich klinisch weiterhin ein aufgetriebener, aber schmerzloser Unterkiefer. Eine erneute *MRT-Bildgebung *zeigte im Vergleich zur Voruntersuchung eine deutliche *Befundbesserung* mit Rückbildung der Umgebungsreaktionen, insbesondere auch der Knochenödeme, sowie die Kontrastmittel(KM)-Anreicherungen betreffend, im allerdings weiterhin aufgetriebenen Unterkiefer rechtsseitig.

Von *Juni 2023 *bis *Juli 2025* – anfänglich parallel zur Pamidronat-Intervall-Therapie – wurde die Therapie mit Adalimumab mit 40 mg s.c., zunächst alle 2 Wochen fortgesetzt, während bereits ab April 2023 eine Streckung der Bisphosphonat-Behandlungen auf 6‑ bzw. 8‑wöchentliche Intervalle bis September 2023 erfolgte und danach nur noch eine einmalige Gabe im März 2024 veranlasst wurde.

Zu diesem Zeitpunkt war die Patientin – von leicht vermehrtem Haarausfall abgesehen – beschwerdefrei, es bestanden keine Kieferschmerzen, es traten weder Infekte noch Fieber auf, die Mundöffnung und das Kauvermögen waren wieder ohne Einschränkungen. Allerdings beobachteten wir eine Zunahme der Akne im Gesichtsbereich, daher verordneten wir befristet erneut Azithromycin 500 mg p.o. täglich, ohne jedoch eine effektive Verbesserung des Hautbefundes festzustellen.

Eine *MRT-Verlaufskontrolle *im *März 2024 *erbrachte den Befund einer deutlichen Abnahme der Osteitis sowie aller radiologischen Inflammationskriterien. Korrelierend zur Klinik war hier somit eine *inflammatorische Remission* anzunehmen, woraufhin die Streckung der Adalimumab-Therapie von 2‑ auf 3‑wöchige Intervalle vereinbart und umgesetzt wurde (Abb. 2).

Im *Juni 2024 *konnte bei einer Dysgnathie sogar die positive Entscheidung zur festsitzenden kieferorthopädischen Behandlungsapparatur getroffen werden, die bis dato insgesamt unkompliziert verlief. Im *November 2024 *und *Juli 2025* erfolgten dann weitere Verlaufskontrollen der inzwischen 18-jährigen Patientin, die – von gelegentlicher Druckschmerzhaftigkeit des Kieferknochens infolge des Tragens der kieferorthopädischen Apparatur abgesehen – vollständig beschwerdefrei blieb. Anamnestisch und klinisch waren hier weder Gesichtsschwellungen noch wesentliche Akne-Effloreszenzen oder eine palmoplantare Pustulosis zu eruieren. Zu keiner Zeit beobachteten wir eine Hyperostosis sternocostoclavicularis oder eine periphere Synovitis. Stattgehabte Infekte waren nicht zu verzeichnen.

Auch eine vorläufig letzte *MRT-Kontrolle am 30.10.2025* (Abb. 1a) zeigte eine *Remission* der anhaltend beschwerdefreien, inzwischen erwachsenen Patientin, die vor Monaten eine Ausbildung zur Verwaltungsangestellten aufgenommen hat.

## Kritische Wertung der Therapie vor dem Hintergrund aktueller Konsens-Empfehlungen 2025 [3–5]

Die Krankengeschichte unserer Patientin repräsentiert den langen Leidensweg vom ersten Symptom im ambulanten Bereich bis zur Diagnosestellung der CNO in einem Rheumatologischen Zentrum. Die Erstvorstellung in unserem Klinikum erfolgte in der Klinik für Mund‑, Kiefer- und Gesichtschirurgie. Von dort aus erfolgte die Übernahme in unsere Rheumatologische Klinik.

Die Initialtherapie mit Antibiotika, konventionellen synthetischen Disease-Modifying Anti-Rheumatic Drugs (csDMARD) in Verbindung mit NSAR bzw. Steroiden und oralen Bisphosphonaten zeigten kein adäquates Ansprechen, so dass ein Regime-Wechsel auf das i.v. applizierte Bisphosphonat-Präparat Pamidronat plus Tumornekrosefaktor-α-Inhibitor (TNFi) Adalimumab folgte. Gemäß der AWMF-Leitlinien zur Vermeidung von Kiefernekrosen unter antiresorptiver Therapie erfolgte vorab eine MKG-Untersuchung zum Ausschluss sanierungsbedürftiger Herde [2].

Inwieweit die moderate Akne der jugendlichen Patientin erkrankungsrelevant oder noch als altersentsprechend und physiologisch anzusehen ist, kann diskutiert werden.

Nach Umstellung der Behandlungsstrategie konnte ein nachhaltig positives Therapieansprechen mit sowohl klinischer als auch radiologischer Remission dokumentiert werden. Wenngleich grundsätzlich ein später Effekt der zeitlich zurückliegenden Therapien sowie eine spontane Befundbesserung nicht absolut ausgeschlossen werden können, ist die initiale Therapie mit konventionellen csDMARD aus unserer Sicht als nicht hinreichend effektiv zu bewerten.

Adalimumab wurde zuletzt nur noch 1‑mal monatlich appliziert, die Bisphosphonat-Applikationsintervalle wurden ebenfalls deutlich zeitlich gestreckt, die Behandlung konnte inzwischen beendet werden. Auch unter diesem Therapiemanagement trat weiterhin kein *Relapse* auf, sodass die im Krankheitsverlauf verabreichte Kombinationstherapie bei ausgeprägt inflammatorischer Aktivität hier plausibel effektiv erscheint.

Oral applizierte Bisphosphonate unterliegen einer sehr inkonstanten Resorptionskinetik, daher sind intravenös zu applizierende Bisphosphonate klar zu favorisieren. Die größte Expertise bei CNO in der Adoleszenz bzgl. der Nutzen-Risiko-Abwägung findet sich hinsichtlich der Anwendung von Pamidronat i.v. in einer Dosierung von 60 mg pro Applikation. Osteonekrosen des Kiefers sind eine mögliche Komplikation dieser Anwendung, treten bei dieser Indikation und hier angewandter Dosierung und bei guter Mundhygiene sowie zurückhaltend angewandter oralchirurgischer Maßnahmen nur sehr selten auf.

Unter Berücksichtigung und Kenntnis der inzwischen vorhandenen Konsens-Empfehlungen, sowohl die juvenile aber auch die adulte CNO betreffend, kann bei Vorhandensein entsprechender Expertise zukünftig eine präzise Behandlungsstrategie in Form einer Mono- oder Kombinationstherapie aus TNF-α-Inhibitoren und/oder Bisphosphonaten mit guten Behandlungsaussichten Anwendung finden [3–5].

Antibiotikabehandlungen – wie auch in unserem Fall zunächst noch angewandt – haben hier inzwischen keine Bedeutung mehr, sind vielmehr als obsolet anzusehen.

Unsere Kasuistik zeigt die Herausforderungen in der Therapie der juvenilen CNO auf und unterstützt die aktuellen Empfehlungen, welche moderne medikamentöse Behandlungsstrategien priorisieren. Die Implementierung regelmäßiger Reevaluationen, auch mittels MRT, für die gewünschte Treat-to-Target-Strategie ist dabei essenziell und insbesondere auch bei jungen Patientinnen und Patienten im Gegensatz zur CT sicher und strahlenfrei anwendbar.

Weitere Therapiekonzepte und neue vielversprechende therapeutische *Targets* müssen im Rahmen von möglichst prospektiven Studien ihre Einordnung finden.

## Fazit

Unser Fallbeispiel zeigt die CNO-Behandlungssituation im Übergang vom Jugend- zum Erwachsenenalter, die von einem hohen Leidensdruck geprägt ist, inzwischen aber durch aktuelle Behandlungsstrategien gut beherrschbar und mittels MRT zuverlässig und strahlungsfrei beurteilbar ist.
